# Supplementary material for: Renal Medullary Carcinomas Harbor a Distinct Methylation Phenotype and Display Aberrant Methylation of Genes Related to Early Nephrogenesis
Source: Cancers (Basel). 2022 Oct 14;14(20):5044. doi: 10.3390/cancers14205044 (PMC9599670; doi:10.3390/cancers14205044)
Supplement: Supplementary file 1 [file cancers-14-05044-s001.zip › Figure S1.pdf]

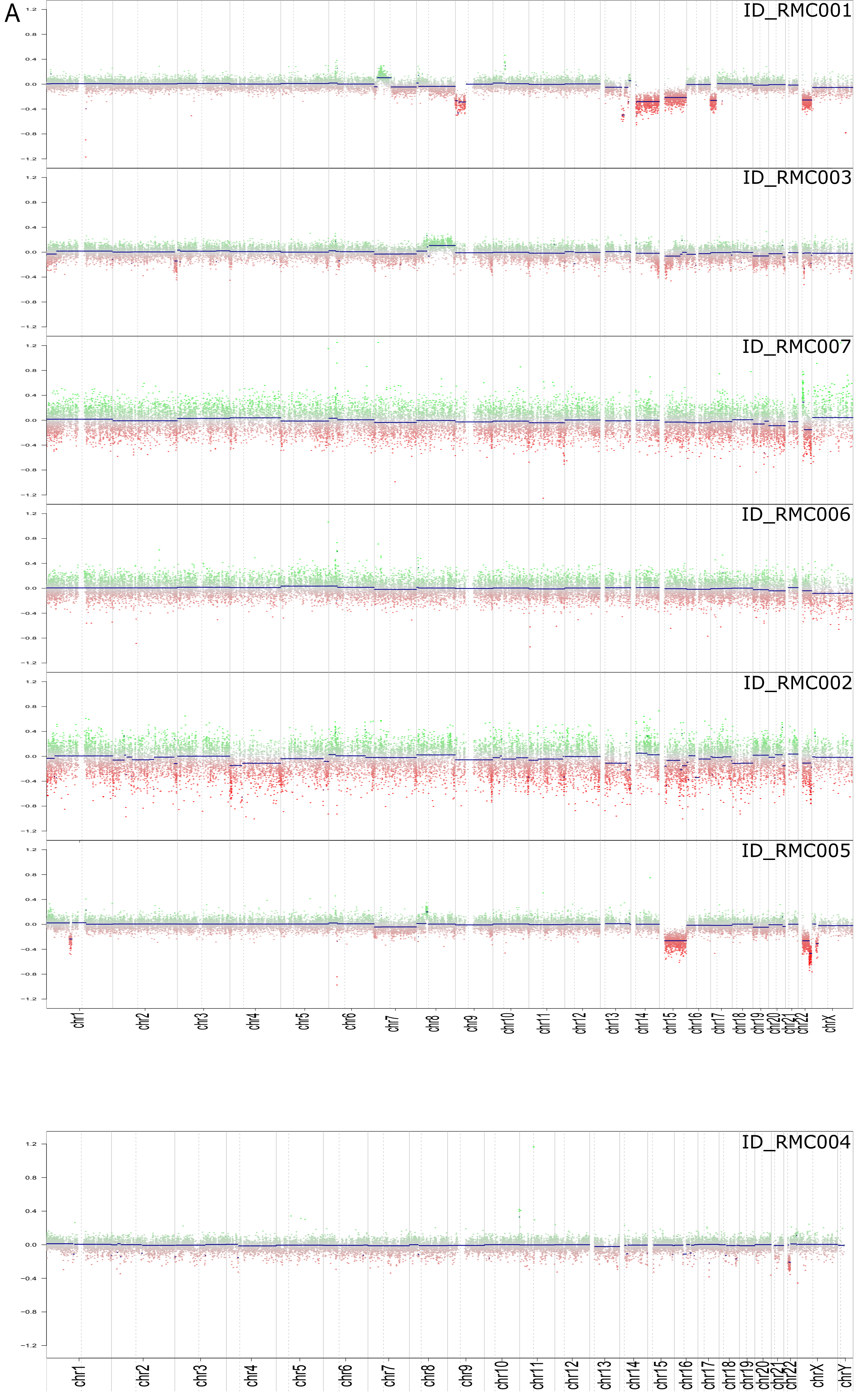
A
ID_RMC001
ID_RMC003
ID_RMC007
ID_RMC006
ID_RMC002
ID_RMC005
ID_RMC004
1.2
0.8
0.4
0.0
−0.4
−0.8
−1.2
chr1
chr2
chr3
chr4
chr5
chr6
chr7
chr8
chr9
chr10
chr11
chr12
chr13
chr14
chr15
chr16
chr17
chr18
chr19
chr20
chr21
chr22
chrX
chrY

B

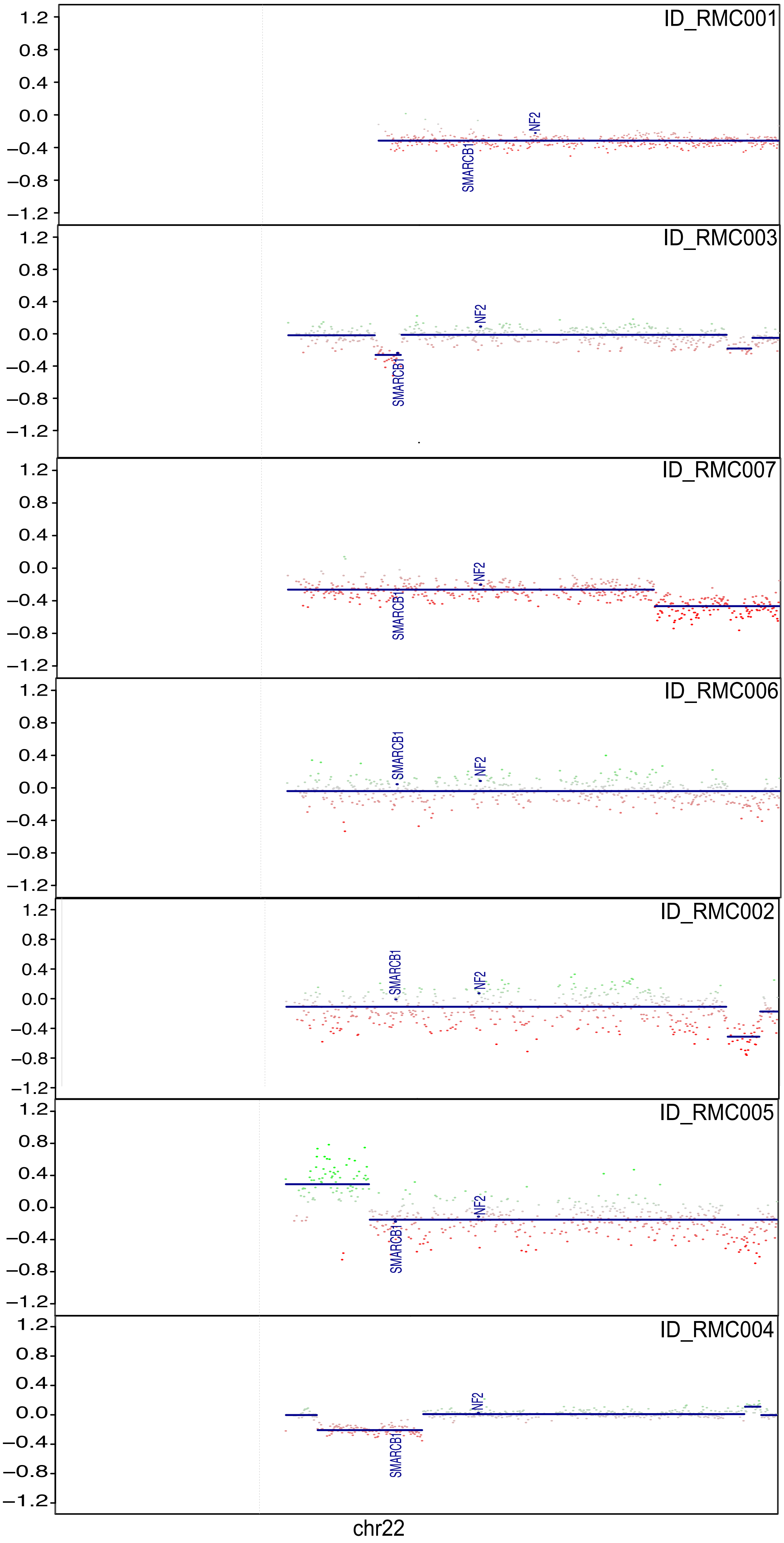

Supplemtary Figure S1: Copy number profiles from all RMC pateints alaysed in this study. (A) Copy number profiles for all chromosomes. (B) Copy number profiles for chr22.
