# Supplementary material for: Renal Medullary Carcinomas Harbor a Distinct Methylation Phenotype and Display Aberrant Methylation of Genes Related to Early Nephrogenesis
Source: Cancers (Basel). 2022 Oct 14;14(20):5044. doi: 10.3390/cancers14205044 (PMC9599670; doi:10.3390/cancers14205044)
Supplement: Supplementary file 1 [file cancers-14-05044-s001.zip › Figure S3.pdf]

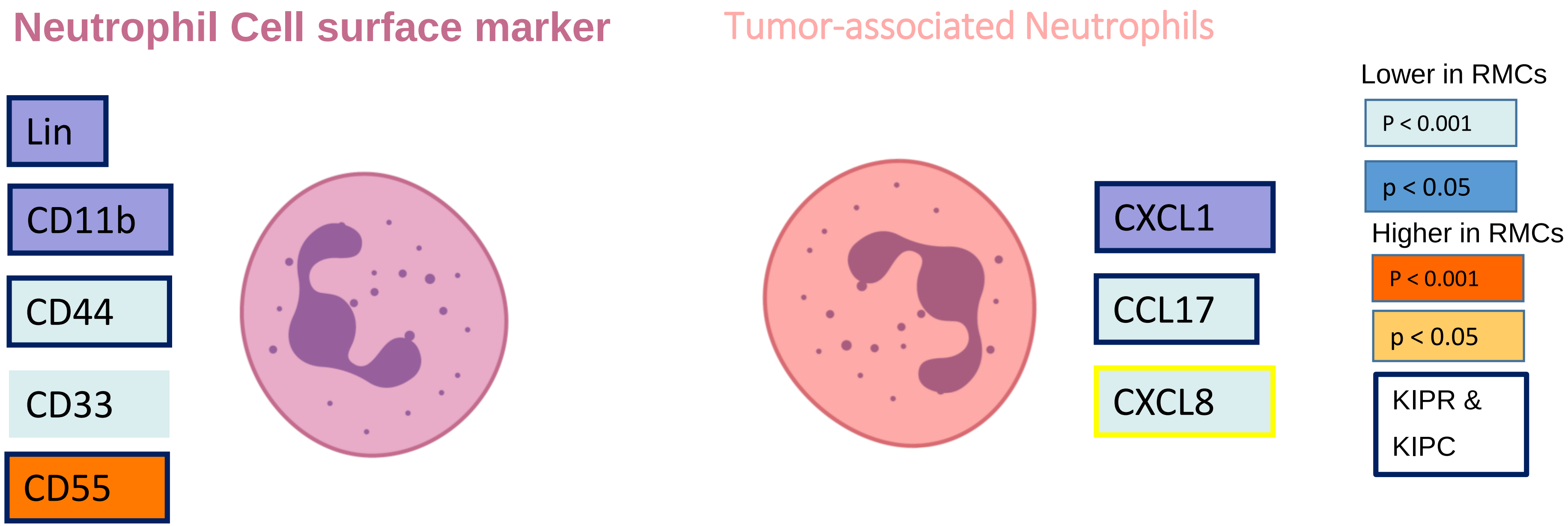

Supplementary figure S3: Differentally methylated genes encoding for neutrophil cell surface markers and markers for tumor-associated neutrophils im RMCs compared to KIPRs and KIPCs.
