# Supplementary material for: Renal Medullary Carcinomas Harbor a Distinct Methylation Phenotype and Display Aberrant Methylation of Genes Related to Early Nephrogenesis
Source: Cancers (Basel). 2022 Oct 14;14(20):5044. doi: 10.3390/cancers14205044 (PMC9599670; doi:10.3390/cancers14205044)
Supplement: Supplementary file 1 [file cancers-14-05044-s001.zip › Figure S5.pdf]

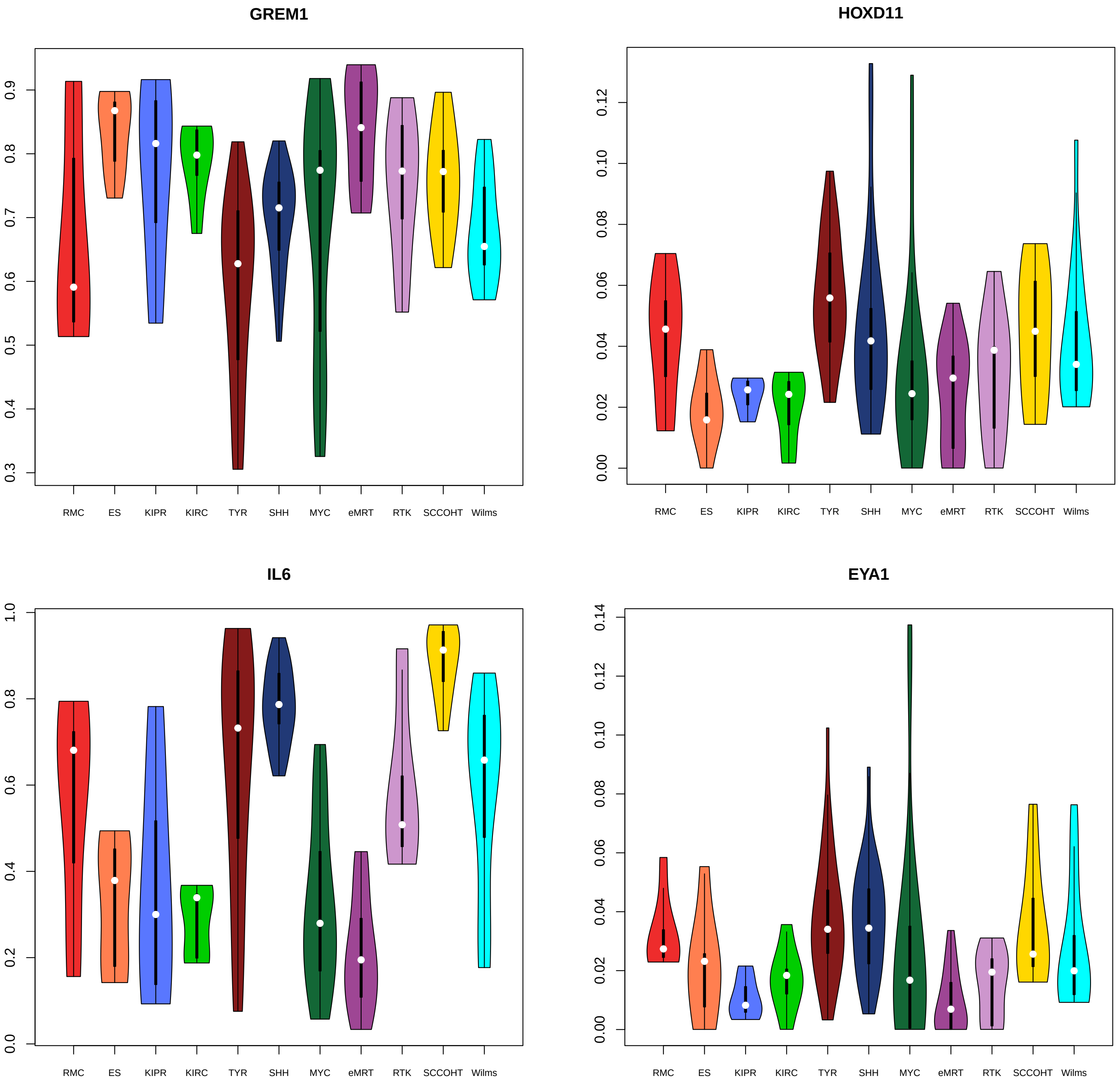

Supplementary Figure S5: Violin Plots displaying exemplary genes, that are significantly different methylated in RMCs compared to ES in the top row (GREM1 - ES mean = 0.833, RMC mean = 0.67, p = 0.0482; HOXD11 - ES mean = 0.0191, RMC mean = 0.0426, p = 0.029) and genes without significant differences in the bottom row (IL6 - ES mean = 0.325, RMC mean = 0.5597, p = 0.061; EYA1 - ES mean = 0.021, RMC mean = 0.0322, p = 0.21)
