# Supplementary material for: Renal Medullary Carcinomas Harbor a Distinct Methylation Phenotype and Display Aberrant Methylation of Genes Related to Early Nephrogenesis
Source: Cancers (Basel). 2022 Oct 14;14(20):5044. doi: 10.3390/cancers14205044 (PMC9599670; doi:10.3390/cancers14205044)
Supplement: Supplementary file 1 [file cancers-14-05044-s001.zip › Supplementary Table S1.pdf]

Supplementary Table S1

Renal medullary carcinoma cases and corresponding IDAT files

ID (Jia et al., 2019)	IDAT	Patho ID	IDs for cases from this study
	204375420076_R02C01	S17_514_3	ID_RMC001
	204375420079_R08C01	S19_13485	ID_RMC002
	204375420079_R06C01	S20_20897	ID_RMC003
	203033970041_R01C01	I123_017	ID_RMC004
Case #6	204375420088_R02C01	S18_16454	ID_RMC005
Case #2	204375420079_R07C01	S07_3938	ID_RMC006
Case #9	204375420088_R01C01	S06_1616G	ID_RMC007
